# (Pro)renin Receptor-Dependent Induction of Profibrotic Factors Is Mediated by COX-2/EP4/NOX-4/Smad Pathway in Collecting Duct Cells

**DOI:** 10.3389/fphar.2019.00803

**Published:** 2019-07-23

**Authors:** Cristian Reyes-Martinez, Quynh My Nguyen, Modar Kassan, Alexis A. Gonzalez

**Affiliations:** ^1^Instituto de Química, Pontificia Universidad Católica de Valparaíso, Valparaiso, Chile; ^2^Skaggs School of Pharmacy and Pharmaceutical Sciences, University of California, San Diego, San Diego, CA, United States; ^3^Cardiovascular Division, Department of Medicine, Abboud Cardiovascular Research Center, University of Iowa Carver College of Medicine, Iowa City, IA, United States

**Keywords:** (Pro)renin receptor, cyclooxygenase inhibition, reactive oxygen species, intrarenal renin–angiotensin system, collecting duct renin

## Abstract

The binding of prorenin to the (pro)renin receptor (PRR) triggers the activation of MAPK/ERK1/2 pathway, induction of cyclooxygenase-2 (COX-2), NOX-4-dependent production of reactive oxygen species (ROS), and the induction of transforming growth factor β (TGF-β) and profibrotic factors connecting tissue growth factor (CTGF) and plasminogen activator inhibitor (PAI-I) in collecting duct (CD) cells. However, the role of COX-2 and the intracellular pathways involved are not clear. We hypothesized that the PRR activation increases profibrotic factors through COX-2-mediated PGE2 activation of E prostanoid receptor 4 (EP4), upregulation of NOX-4/ROS production, and activation of Smad pathway in mouse CD cells. Recombinant prorenin increased ROS production and protein levels of CTGF, PAI-I, and TGF-β in M-1 CD cell line. Inhibition of MAPK, NOX-4, and COX-2 prevented this effect. Inhibition of MEK, COX-2, and EP4 also prevented the upregulation of NOX-4. Because TGF-β activates Smad pathway, we evaluate the phosphorylation of Smad2 and 3. COX-2 inhibition or EP4 antagonism significantly prevented phosphorylation of Smad 2/3. Mice that were infused with recombinant prorenin showed an induction in the expression of CTGF, PAI-I, TGF-β, fibronectin, and collagen I in isolated collecting ducts as well as the expression of alpha smooth muscle actin (α-SMA) in renal tissues. COX-2 inhibition prevented this induction. These results indicate that the induction of TGF-β, CTGF, PAI-I, and ROS occurs through PRR-dependent activation of MAPK and NOX-4; however, this mechanism depends on COX-2-derived PGE2 production and the activation of EP4 and Smad pathway.

## Introduction

The binding of prorenin to the (pro)renin receptor (PRR) triggers the phosphorylation of mitogen-activated protein kinases/extracellular regulated kinases 1/2 (MAPK/ERK1/2) (Batenburg et al., 2007; Feldt et al., 2008; Muller et al., 2008; Ballarin-Gonzalez et al., 2013) and upregulates cyclooxygenase-2 (COX-2) in kidney tissues (Kaneshiro et al., 2006). We have reported that PRR activation by recombinant prorenin increases COX-2 expression independently of angiotensin (ANG) II in cultured renal collecting duct (CD) cells (Gonzalez et al., 2013). Both activation and upregulation of PRR have been associated with renal tissue damage (Kaneshiro et al., 2006; Kaneshiro et al., 2007; Ichihara et al., 2008). Liu showed that proximal tubular cells treated with prorenin show upregulation of transforming growth factor beta (TGF-b1) and alpha-smooth muscle actin (α-SMA) (Yisireyili et al., 2014). In human kidney embryonic (HEK) cells, augmentation of reactive oxygen species (ROS) is observed after PRR stimulation. This effect is mediated by a NOX-4-dependent mechanism (Clavreul et al., 2011).

We recently showed that cultured CD cells treated with nanomolar concentrations of recombinant prorenin undergo to epithelial–mesenchymal transition and have increased levels of intracellular ROS, activation of MAPK pathway, and upregulation of profibrotic factors including CTGF, plasminogen activator inhibitor-1 (PAI-I) and TGF-β, fibronectin, and collagen I (Gonzalez et al., 2017). Although there are still a discrepancy between the plasma levels of prorenin and the effective physiological concentrations for PRR activation in the kidney (Campbell et al., 2009), it has been shown that high plasma prorenin is present in patients with diabetic nephropathy (Franken et al., 1992), a condition that is associated with microvascular pathologies (Chiarelli et al., 2001). PRR contributes to development of diabetic kidney disease through TGF-β and connective tissue growth factor (CGTF) signaling cascade (Huang et al., 2011). Yoshida et al. demonstrated that high plasma prorenin plays a role in the development of coronary artery disease (Yoshida et al., 2015). Interestingly, it has been shown that African-Americans who have known susceptibility to high blood pressure showed disproportionately high levels of prorenin (Tu et al., 2012).

In diabetic animal models, there is an increase in prorenin and renin expression in the CD (Kang et al., 2008). Because PRR is expressed in the neighbor CD intercalated cell (Gonzalez et al., 2013), prorenin or renin coming from the principal cells of the CD might stimulate PRR, leading to activation of signaling pathways such as MAPK/ERK 1/2 and induction of COX-2. Although the events that follow the activation of PRR on ROS generation, MAPK pathway activation, and upregulation of profibrotic genes have been partially described (Clavreul et al., 2011; Gonzalez et al., 2017), the role of COX-2 in this regulation is not fully understood. It has been shown that the ERK1/2 pathway and cAMP/PKA pathway increase the expression of NOX-4 (Clavreul et al., 2011; Muzaffar et al., 2012). Activation of the Gs (cAMP/PKA) coupled prostaglandin receptor EP4 increases NOX-4 expression in liver cells. In addition, overexpression of COX-2 shows higher NOX4 levels and ROS content, while the presence of a COX-2 inhibitor decreases these effects (Sancho et al., 2011). Reactive oxygen species (ROS) are involved in TGF-β and Smad signaling (Lafon et al., 1996; Hong et al., 1997; Chiu et al., 2001), which are known to be activators of fibrotic factors such as CTGF and PAI-I (Clarkson et al., 1999; Kilari et al., 2018). The ERK pathway can enhance Smad activity. Additionally, ERK inhibition reduces TGF-β1-stimulated Smad phosphorylation as well as collagen production and promoter activities, suggesting that ERK activity is necessary for an optimal response to TGF-β1 (Hayashida et al., 2003).

In the present study, we aimed to demonstrate that the activation of PRR increases profibrotic factors through COX-2-mediated PGE_2_ activation of E prostanoid receptor 4 (EP4), the upregulation of NOX-4/ROS production, and activation of Smad pathway in mouse CD cells. To test this, M-1 CD cell line was treated with recombinant prorenin with and without inhibition of MAPK pathway, NOX-4 and COX-2. Specific pharmacological blockade of EP4 receptor was also tested in M-1 cells incubated with hrPR. Since Smad 2 and 3 are considered as downstream mediators of TGF-β signaling (Meng et al., 2015) and because TGF-β is induced by ROS, we evaluated the phosphorylation of Smad2 and 3, which are activated by TGF-β receptor in the presence of EP4 antagonist. Additionally, we performed *in vivo* experiments in mice infused with human recombinant prorenin (100 ng/min) *via* an osmotic minipump for 36 h with and without selective COX-2 inhibitor. The expression of profibrotic factors was analyzed in isolated CDs and renal medullary tissues.

## Materials and Methods

### M-1 Cell Culture

M-1 cells (ATCC, VA) are a CD cell line with phenotypic characteristics of cortical CD cells (Stoos et al., 1991). M-1 cells are composed of principal cells and intercalated cells constitutively expressing COX-2 (Nasrallah et al., 2001), prorenin–renin, and PRR constitutively (Gonzalez et al., 2015; Gonzalez et al., 2017; Gonzalez et al., 2017). The M-1 cells were cultured as previously described (Gonzalez et al., 2015; Gonzalez et al., 2016; Gonzalez et al., 2017). Cells were harvested after 6 h of treatments with human recombinant prorenin (hrPR) (Cayman Chemical, EE.UU). at 10^−8^ mol/l according to its described range of affinity in nanomolar range (Batenburg et al., 2007; Wilkinson-Berka, 2008)

### Pharmacological Blockers in M-1 Cells

Treatment with NOX-4 inhibitor GKT 137831 was performed at three different concentrations (10, 20, and 30 µM) to explore the effect on ROS production according to the literature (Sedeek et al., 2013a). GKT 137831 was then used at 30 µM for protein expression analysis. Similarly, we tested the effects of PD98059, a potent and selective inhibitor of MAP kinase kinases (MAPKK), MEK1 and MEK2 (Alessi et al., 1995) at two concentrations (30 and 50 µM) (Gonzalez et al., 2017), to explore the effects on ROS production and induction of profibrotic proteins mediated by hrPR. NS-398 was used at 10^−5^ mol/l (Ferguson et al., 1999) to determine COX-2 inhibition effect on ROS and profibrotic protein expression. CD cells show high expression of EP4 receptors (Gonzalez et al., 2013; Wang et al., 2016). We used L-161982 (Cayman Chemical), a potent and selective EP4 receptor antagonist that demonstrates selective binding to human EP4 receptors with a Ki value of 0.024 M. We used a fourfold higher concentration (100 nM) (Takayama et al., 2002). All pharmacological inhibitors were added 30 min before incubations with hrPR. M-1 CD cells were harvested after 6 h. Controls were performed with vehicle (DMSO, 0.06% vol/vol).

### Measurement of Reactive Oxygen Species in M-1 Cells

M-1 cells were seeded in 96-well black polystyrene plates and treated with MEK, COX-2, or NOX-4 inhibitors during 15 min at 37°C. Then, all groups were treated with probe carboxy-2′, 7′-dichloro-dihydro-fluorescein diacetate (DCFHDA, Sigma Chemical Co, St. Louis, MO, USA) at 25 µM for 30 min at 37°C. Fluorescence measurements of DCF (the product of H2DCFDA oxidation: excitation, 495 nm; emission, 529 nm) were performed on a plate reader (Appliskan; Thermo Fisher Scientific, Waltham, MA, USA). To normalize results, total protein from each well was quantified by the bicinchoninic acid (BCA) method. A positive control was conducted using 50 µM H_2_O_2_.

### *In Vivo* Treatments

The Institutional Animal Care and Use Committees approved all animal protocols. Male CF-1 mice (18–20 g, *n* = 5) were cage housed and maintained in a temperature-controlled room with 12-h light/dark cycles with free access to tap water and standard rat chow. Experiments with chronic infusions of prorenin have been performed previously in rhesus monkeys at 400 ng/min, causing three- to fourfold increases in normal plasma prorenin concentrations from ∼70 to ∼250 ng/ml/h (Lenz et al., 1990). High plasma prorenin, as high as ∼1,000 pg/ml, has been found in patients with cardiovascular risk (Yoshida et al., 2015). Human recombinant prorenin (Cayman Chemicals) was infused at a rate of 100 ng/min *via* osmotic minipump for 36 h. Selective COX-2 inhibitor NS-398 attenuates myocardial fibrosis in mice at 5 mg kg (Chi et al., 2017) and is able to block the LPS-induced increase in PGE_2_ in rats at same dose (Lugarini et al., 2002). NS-398 (Cayman Chemicals, EE.UU). was administered in 5% aqueous methylcellulose solution by oral gavage every 6 h. Sham-operated mice were used as controls and administered methylcellulose solution. For physiological parameters presented in **Table 1**, four mice were placed in cages for urine collections. Urine osmolality was measured by vapor pressure osmometry (Vapro Osmometer, model 5600, Wescor). Creatinine measurements in plasma and urine were used to calculate the estimated creatinine clearance over 16 h as an approach to determine renal function. Urinary sodium and potassium were measured as described previously (Gonzalez et al., 2014).

**Table 1:** Physiological parameters in mice after 36 h of subcutaneous infusions with saline, human recombinant prorenin (hrRP), and hrPR plus COX-2 inhibitor NS-398.

	Saline	hrPR	NS398	hrPR+NS398
Body weight, g	22 ± 3	20 ± 3	18 ± 4	19 ± 4
Kidney weight, g	0.29 ± 0.01	0.30 ± 0.02	0.28 ± 0.03	0.29 ± 0.01
Urine osmolality, mosmol/kgH2O	345 ± 8	375 ± 9*	348 ± 8	380 ± 8*
Urine flow, ml/16 h	6.02 ± 0.02	5.35 ± 0.05	4.91 ± 0.06	4.21 ± 0.10*
Estimated GFR, ml·min^−1^	0.92 ± 0.03	0.89 ± 0.04	0.86 ± 0.05	0.84 ± 0.05*
FENa %	0.91 ± 0.02	0.88 ± 0.02	0.87 ± 0.02	0.87 ± 0.03
FEK %	1.4 ± 0.2	1.3 ± 0.3	1.4 ± 0.4	1.3 ± 0.2

*p <0.05 versus Saline group.

### Immunofluorescence in Freshly Isolated Collecting Ducts

At the end of the study, mice were euthanized by conscious decapitation, and renal tissues were collected to perform immunofluorescence and Western blots in freshly isolated inner medullary collecting ducts. Freshly isolated collecting ducts were prepared as previously described (Gonzalez et al., 2011), with variations in digestion time and wash steps. Briefly, inner medullary tissues were digested in 10 ml of DMEM-Ham F-12, 20 mg of collagenase B, 7 mg of hyaluronidase, 80 mmol/l of urea, and 130 mmol/l of NaCl and incubated at 37°C under continuous agitation for 30 mi. After centrifugation, the pellet was washed in prewarmed culture medium without enzymes. The resulting IMCD cell suspension was seeded in six-well chambers (Nalge Nunc, Rochester, NY, USA) and fixed in cold methanol for 20 min, blocked with PBS-Tween (0.1%) plus BSA (3%) for 1 h, and stained with anti-fibronectin (Cat. No. sc-8422; Santa Cruz Biotechnology) or anti-collagen I antibody (Cat. No. 34710, Abcam) at 1:200 dilutions and detected with secondary antibody Alexa Fluor 488 conjugated to anti-rabbit IgG (Invitrogen) at 1:500 dilutions. Negative controls were obtained by omission of the specific primary antibody. Measurements of fluorescence intensity were performed with NIS Elements software (Nikon) in 10 fields from each processed kidney and expressed as fluorescence intensity versus total number of collecting duct in each field previously counted in light field (**Figure 5B**).

### Tissue Immunofluorescence

Kidney sections (3 μm) were stained with rabbit anti-PRR (Cat. No. HPA003156, Sigma Chemical Co, St. Louis, MO, USA) at 1:200 dilutions or α-SMA antibody from Abcam (ab5694, Abcam, Cambridge, MA, USA) followed by the incubation of the corresponding immunofluorescent secondary antibody (1:1,000, Alexa Fluor^®^ 594, Invitrogen, Carlsbad, CA, USA). Negative controls were obtained by omission of the specific primary antibody. Samples were counterstained with 4′,6-diamidino-2-phenylindole (DAPI, Invitrogen, Carlsbad, CA, USA) for nuclei staining.

### Protein Expression Analysis

Forty micrograms of total protein were used for Western blot analysis. Protein expression levels were quantified after immunoblotting using a 1:1,000 dilution of the following specific antibodies: connecting tissue growth factor (CTGF; Cat. No. sc-25440, Santa Cruz Biotechnology), PAI-1 (Cat. No. SC-8979, Santa Cruz Biotechnology), TGF-β (Cat. No. SC-130348, Santa Cruz Biotechnology), COX-2 antibody (Cayman, Ann Arbor, MI, USA), mouse anti-phospho-p44/42 ERK1/2 (Thr202/Tyr204), and a rabbit anti-total ERK antibody (Cell Signaling Technology, Beverly, MA, USA). NOX-4 antibody was purchased from Santa Cruz (sc-21860). Antibodies against Anti-Smad2/3 antibody and anti p-Smad2/3 were obtained from Abcam (Abcam, Cambridge, MA, USA). Primary antibodies were followed by incubation with either donkey anti-rabbit or anti-mouse IgG IRDye 800 CW (Santa Cruz Biotechnology) at 1:3,000 dilutions. Resulting bands were compared to molecular weight standards (M. Biosources, San Diego, CA, USA). Densitometry was performed with ImageJ software and normalized to monoclonal anti-β-actin antibody (Cat. A2228, Sigma Chemical Co, St. Louis, MO, USA).

### Statistical Analyses

For Western blot, an average number of three to six independent observations was performed for each treatment and represented as fold change versus controls. For *in vivo* studies, five mice were used in each group. Data were evaluated by the Grubb test, followed when appropriate by paired and unpaired Student’s *t*-test or by one-way ANOVA with Tukey post-test. Significance was defined as *p* < 0.05. No significant differences are expressed as “ns”. Results are expressed as mean ± SEM.

## Results

### Recombinant Prorenin Causes ERK1/2 Phosphorylation and Increases COX-2 and NOX-2 Expression in M-1 Cells

**Figure 1:**
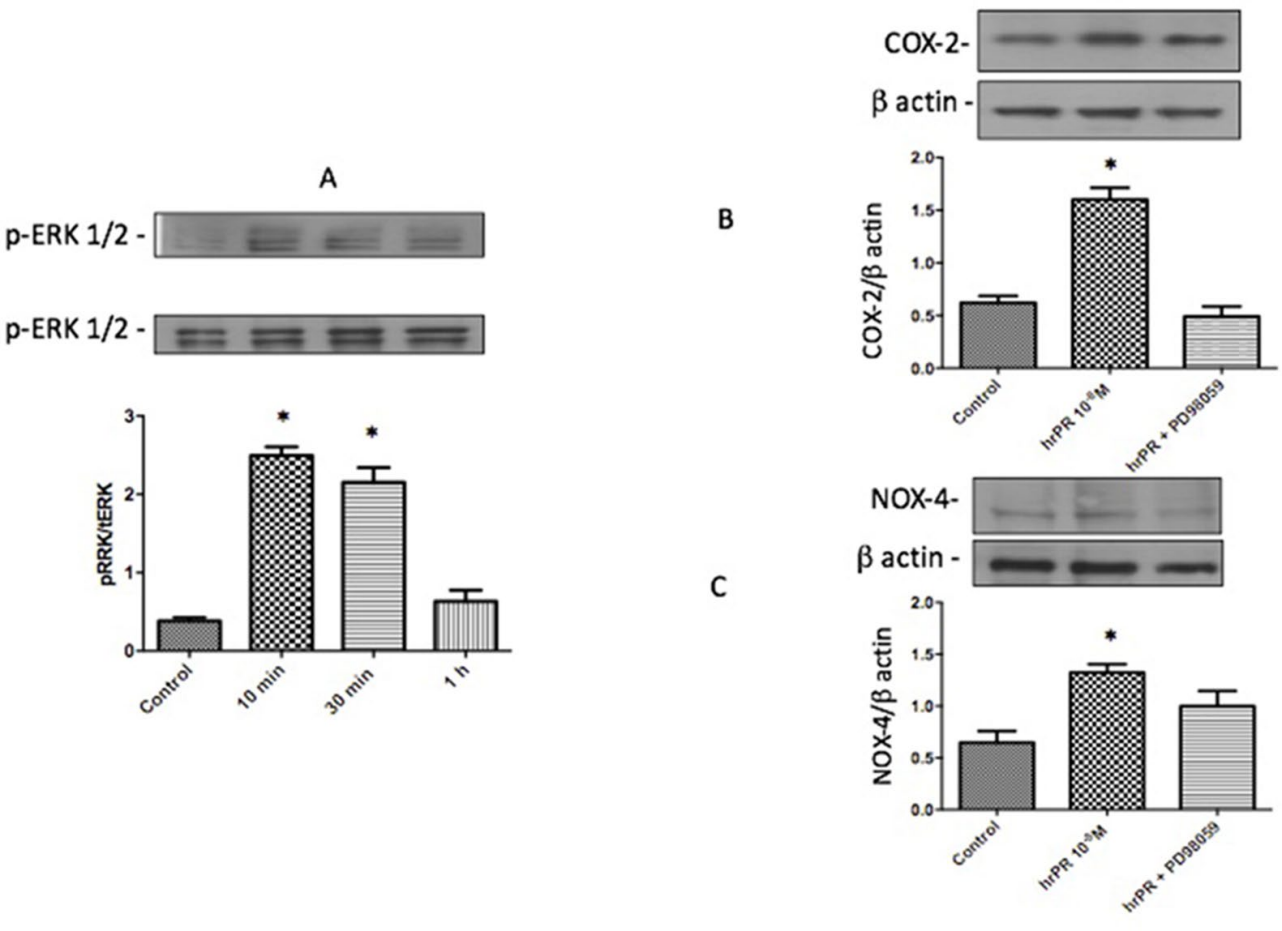
Incubations with recombinant prorenin increase the abundance of COX-2 and NOX-4 *via* MAPK pathway in M-1 cells. **(A)**. Time response of ERK1/2 phosphorylation after incubations with 10^−8^ M human recombinant prorenin (hrPR). MAPK inhibition impairs hrPR-dependent upregulation of COX-2 **(B)** and NOX-4 **(C)**. *p < 0.05, *n* = 4.

As previously described, treatment with recombinant prorenin induced ERK1/2 phosphorylation over the time of incubations, reaching a peak after 10 min of incubation. It subsequently decreased after 1 h (**Figure 1A**). After 6 h, COX-2 and NOX-4 were augmented (ratio protein/β-actin densitometric values: 1.67 ± 0.16 vs. 0.66 ± 0.08 for COX-2 and 1.48 ± 0.08 vs. 0.70 ± 0.19, for NOX4 *p* < 0.05); however, inhibition of MAPK pathway with PD98059 prevented this effect.

### MAPK Inhibition Impairs ROS Formation and the Upregulation of CTGF, TGF-β, and PAI-I Caused by Recombinant Prorenin Incubations

**Figure 2:**
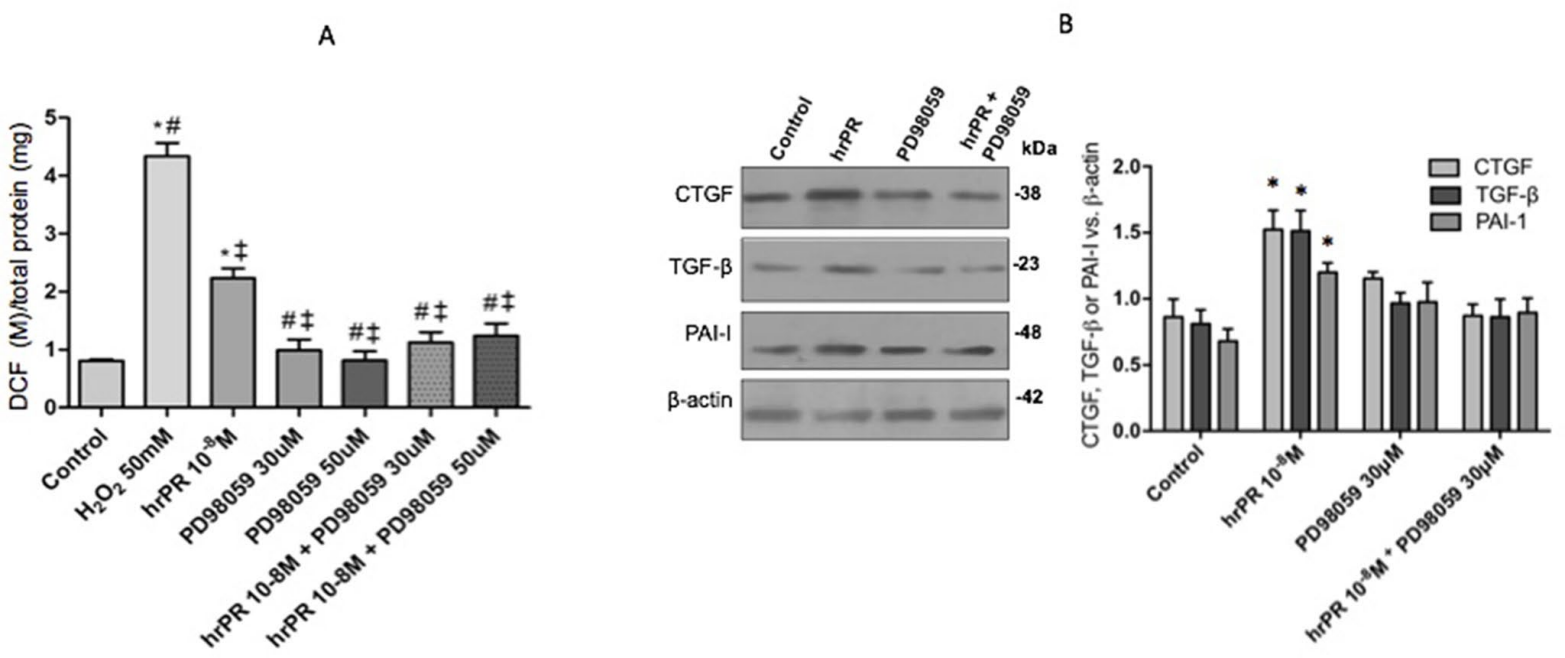
MAPK pathway inhibition by PD98059 prevented the induction of ROS levels and profibrotic markers CTGF, TGF-β, and PAI-I. **(A)**. For ROS quantification, M-1 cells were treated with PD98059 at 30 and 50 µM during 15 min, incubated with DCFH-DA probe for 30 min, and treated with hrPR for 15 min. Results are expressed as DCF probe fluorescence intensity versus total protein (mean ± SEM) **(B)**. Protein levels of CTGF, TGF-β, and PAI-I represented by Western blot analysis (left) and quantitation (right) in M-1 cells incubated during 6 h with hrPR, PD98059, or hrPR plus PD98059, *p < 0.05 versus control, ^#^ *p* < 0,05 versus hrPR group, ^‡^ *p* < 0,05 versus positive control, *n* = 5.

Inhibition of MAPK pathway prevented ROS formation (**Figure 2A**). We tested two concentrations of PD98059: 30 and 50 mmol/l. No differences were found between both concentrations in the blunted effect on hrPR-dependent induction of ROS (control: 0.80 ± 0.01; hrPR: 2.22 ± 0.21, *p* < 0.05 vs. control; PD98059 30 mmol/l: 1.24 ± 0.17, *p* = ns vs. control; PD98059 50 mmol/l: 1.12 ± 0.12, *p* = ns vs. control). Induction of CTGF, TGF-β, and PAI-I was prevented by MAPK inhibition (ratio protein/β-actin densitometric values: 0.87 ± 0.18, 0.86 ± 0.22, 0.90 ± 0.19, respectively, *p* = ns); see **Figure 2B** for immunoblot analysis.

### Induction of ROS and Profibrotic Protein Expression Is Prevented by Inhibition of NOX-4

**Figure 3:**
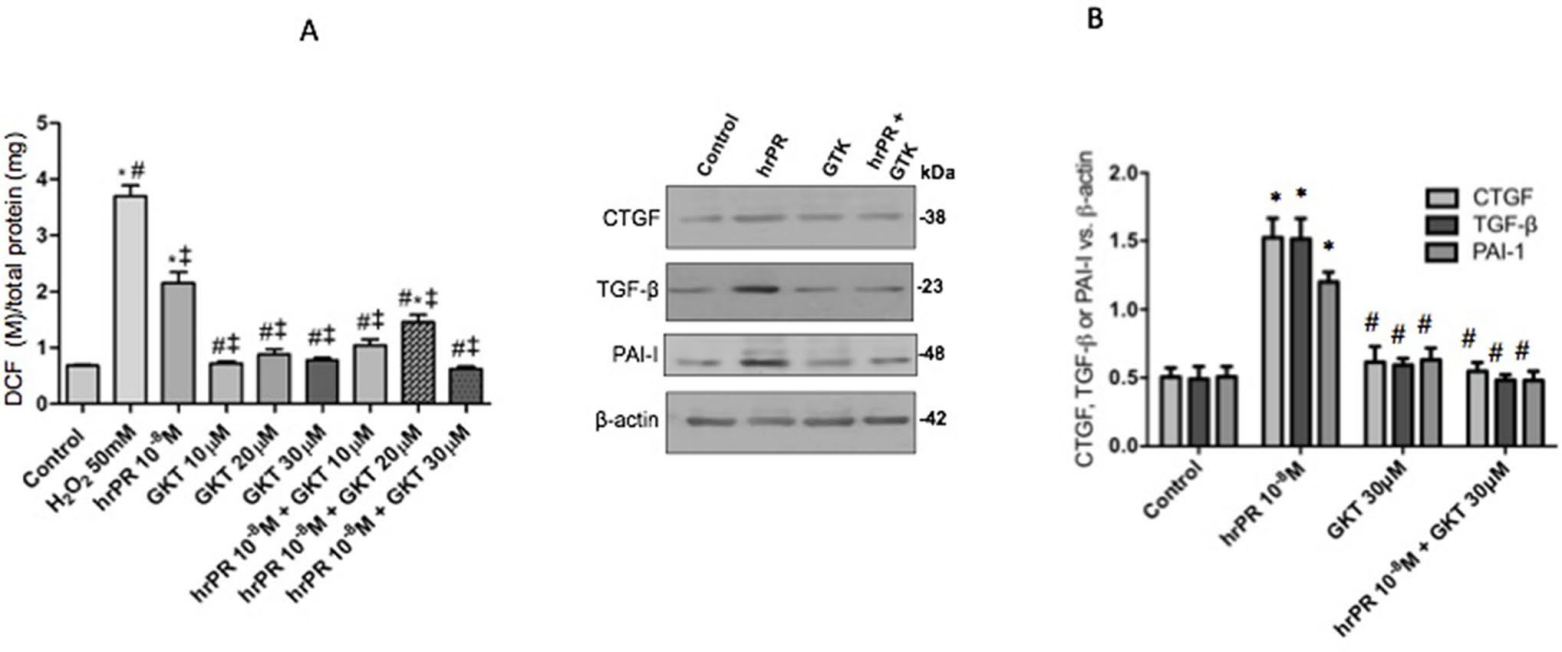
NOX-4 inhibition by pharmacological agent GKT 137831 prevented the induction of ROS and profibrotic markers CTGF, TGF-β, and PAI-I. **(A)**. For ROS quantification, M-1 cells were treated with GKT at 10, 20, and 30 µM during 15 min, incubated with DCFH-DA probe for 30 min, and treated with human recombinant prorenin (hrPR) for 15 min. Results are expressed as DCF probe fluorescence intensity versus total protein (mean ± SEM) **(B)**. Protein levels of CTGF, TGF-β, and PAI-I represented by Western blot analysis (left) and quantitation (right) in M-1 cells incubated during 6 h with hrPR, GTK, or hrPR plus GTK. *p < 0.05 versus control, ^#^ *p* < 0.05 versus hrPR group, ^‡^ *p* < 0.05 versus positive control, *n* = 5.

**Figure 3A** shows the effect of NOX-4 inhibition in M-1 CD cells incubated with human recombinant prorenin (hrPR) at 10^−8^ mol/l. Treatment with hrPR increased DCF/protein ratio (2.15 ± 0.23 vs. 0.68 ± 0.09, *p* < 0.05); however, pre-treatment with GTK 137831 prevented this effect at doses of 30 mmol/l (0.62 ± 0.01 vs. 0.68 ± 0.09, *p* = ns) and to a lesser extent at doses of 10 and 20 mmol/l (1.04 ± 0.15, *p* = ns vs. control and 1.45 ± 0.19, *p* < 0.05 vs. control). Afterward, the following experiments evaluating protein expression were done using 30 mmol/l GTK 137831. **Figure 3B** shows the effects of NOX-4 inhibition on the expression of CTGF, PAI-I, and TGF-β in M-1 cells incubated with hrPR. As observed, hrPR causes a significant increase in protein levels of all three markers analyzed (ratio protein vs. β-actin densitometric values: CTGF, 1.52 ±0.07 vs. 0.50 ± 0.01, *p* < 0.05; TGF-β, 1.51 ± 0.08 vs. 0.49 ± 0.02, *p* < 0.05; PAI-I, 1.21 ± 0.02 vs. 0.50 ± 0.02, *p* < 0.05).

### COX-2 Inhibition Impairs the Induction of ROS NOX-4 and Profibrotic Genes in M-1 CD Cells Treated With Recombinant Prorenin

**Figure 4:**
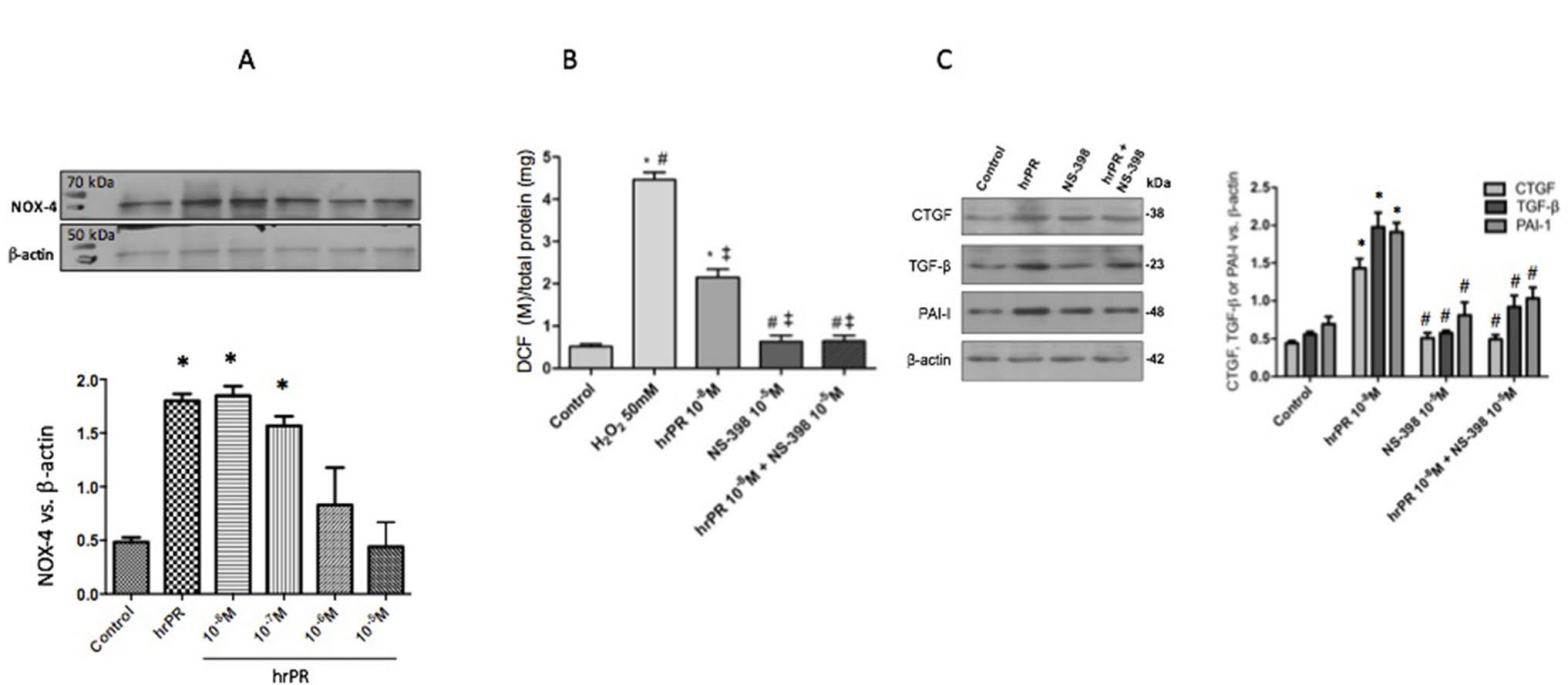
Selective COX-2 inhibitor NS-398 prevented NOX-4 upregulation, the induction of ROS, and the increase in profibrotic markers CTGF, TGF-β, and PAI-I. **(A)**. A dose response effect of COX-2 inhibitor NS-398 on NOX-4 protein expression in M-1 cells incubated with hrPR. **(B)**. For ROS quantification, M-1 cells were treated with NS-398 (10^−5^ M) during 15 min, incubated with DCFH-DA probe for 30 min, and treated with hrPR for 15 min. Results are expressed as DCF probe fluorescence intensity versus total protein (mean ± SEM) **(C)**. Protein levels of CTGF, TGF-β, and PAI-I represented by Western blot analysis (left) and quantitation (right) in M-1 cells incubated during 6 h with hrPR, NS-398, or hrPR plus NS-398, *p < 0.05 versus control, ^#^ *p* < 0.05 versus hrPR group, ^‡^p < 0.05 versus positive control, *n* = 5.

We performed new experiments incubating M-1 cells with hrPR or hrPR plus pre-incubations with specific COX-2 inhibitor NS-398. As shown in **Figure 4A**, NS-398 completely blunted the induction of NOOX-4 protein expression at 10^−5^ M. The increases in intracellular ROS caused by hrPR were also blunted by NS-398 (control: 0.51 ± 0.01; hrPR: 2.15 ± 0.18, *p* < 0.05 vs. control; hrPR+NS-398: 0.64 ± 0.08, *p* = ns vs. control, **Figure 4B**). Similarly, the induction of CTGF, TGF-β, and PAI-I was prevented by MAPK inhibition (ratio protein/β-actin densitometric values: 0.87 ± 0.18, 0.86 ± 0.22, 0.90 ± 0.19, *p* = ns vs. control group, **Figure 4C**).

### EP4 Receptor Antagonism Decreases ROS Production, NOX-4 Expression, Induction of TGF-β, CTGF, and PAI-I, and Activation of the Smad Pathway in M-1 Cells Incubated With hrPR

**Figure 5:**
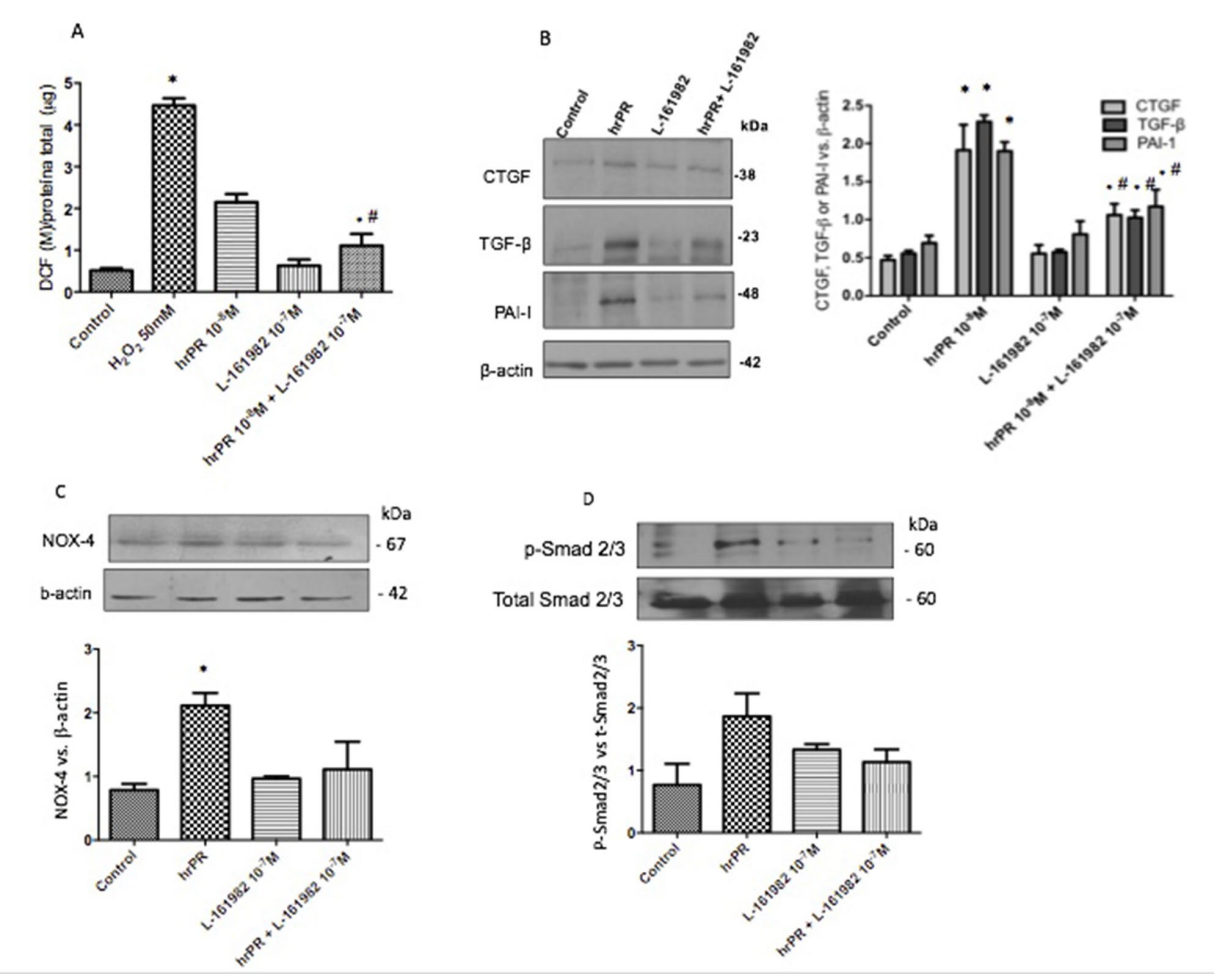
E-Prostanoid receptor 4 blockade prevents hrPR-induced ROS accumulation, NOX-4 upregulation, and induction of profibrotic factors CTGF, TGF-β, and PAI-I. Another set of M-1 cells was tested for ROS production and protein expression of NOX-4, CTGF, TGF-β, and PAI-I. Pre-incubations with 10^7^ M of L-161982 partially prevented ROS induction **(A)** and upregulation of profibrotic factors **(B)**. NOX-4 induction was prevented by EP4 L-161982 **(C)**. Because NOX-4 is responsible for ROS production and TGF-β synthesis, we tested the activation of Smad pathway by examining phosphorylation of Smad2 and 3. Preincubations with EP4 inhibitor prevented Smad2 and 3 phosphorylation levels in response to hrPR incubations **(D)**.

We next tested if pharmacological blockade of the EP4 receptor would be able to prevent ROS formation and the induction of TGF-β, CTGF, and PAI-I in M-1 cells incubated with hrPR. As shown in **Figure 5A**, ROS production was ameliorated in M-1 cells that were treated with hrPR and pre-incubated with L-161982 at 10^−7^ M. The EP4 receptor antagonist partially prevented the induction of TGF-β, CTGF, and PAI-I (**Figure 5B**). This was also associated with a reduction in the protein expression of NOX-4 (**Figure 5C**). EP4 receptor antagonism prevented the phosphorylation of Smad 2/3 (**Figure 5D**), indicating that the activation of TGF-β receptor is involved in the induction of profibrotic factors.

### Chronic Infusion of Recombinant Prorenin Increases the Expression of CTGF, PAI-I, and TGF-β Proteins in Medullary CDs; COX-2 Inhibition Prevents This Effect

**Figure 6:**
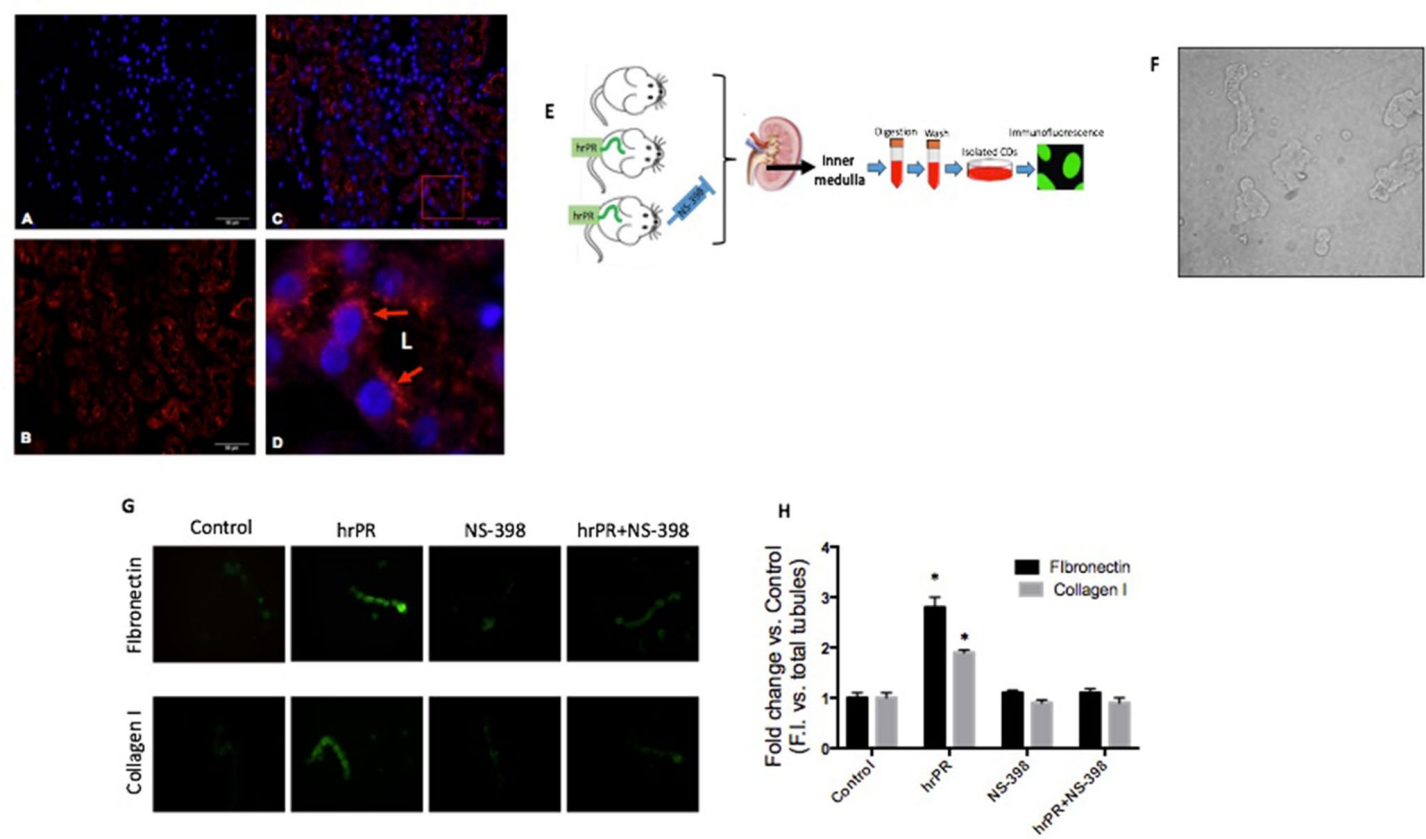
*In vivo* approach to evaluate the effect of chronic hrPR infusion with or without COX-2 inhibition on the expression of collagen I, fibronectin, CTGF, TGF-β, and PAI-I in inner medullary CDs. Immunofluorescence performed in mouse kidney sections showing specific labeling of (Pro)renin receptor in the CDs. **(A)**. DAPI staining for nuclei. **(B)**. Anti-PRR. **(C)** Merge image. **(D)**. 10× digital zoom of merged image showing apical distribution of PRR (red arrows). L indicates tubular lumen. **(E)**. *In vivo* methodology. Human recombinant prorenin was infused at a rate of 100 ng/min *via* an osmotic minipump for 36 h. COX-2 inhibitor NS-398 was administered at 10 mg/kg by oral gavage every 6 h. Sham-operated mice and administered methyl cellulose solution were used as controls. At the end of the study, mice were euthanized by conscious decapitation and renal tissues collected to perform immunofluorescence and Western blots in freshly isolated inner medullary CDs. **(F)**. Representative image of the resulting suspension of freshly isolated inner medullary CDs. **(G)**. Inner medullary CDs were fixed, blocked, and stained with anti-fibronectin or anti-collagen I antibody and detected with secondary antibody Alexa Fluor 488 conjugated to anti-rabbit IgG. **(H)**. Measurements of fluorescence intensity in 10 fields from each processed kidney and expressed as fluorescence intensity versus total number of CD in each field.

**Figure 7:**
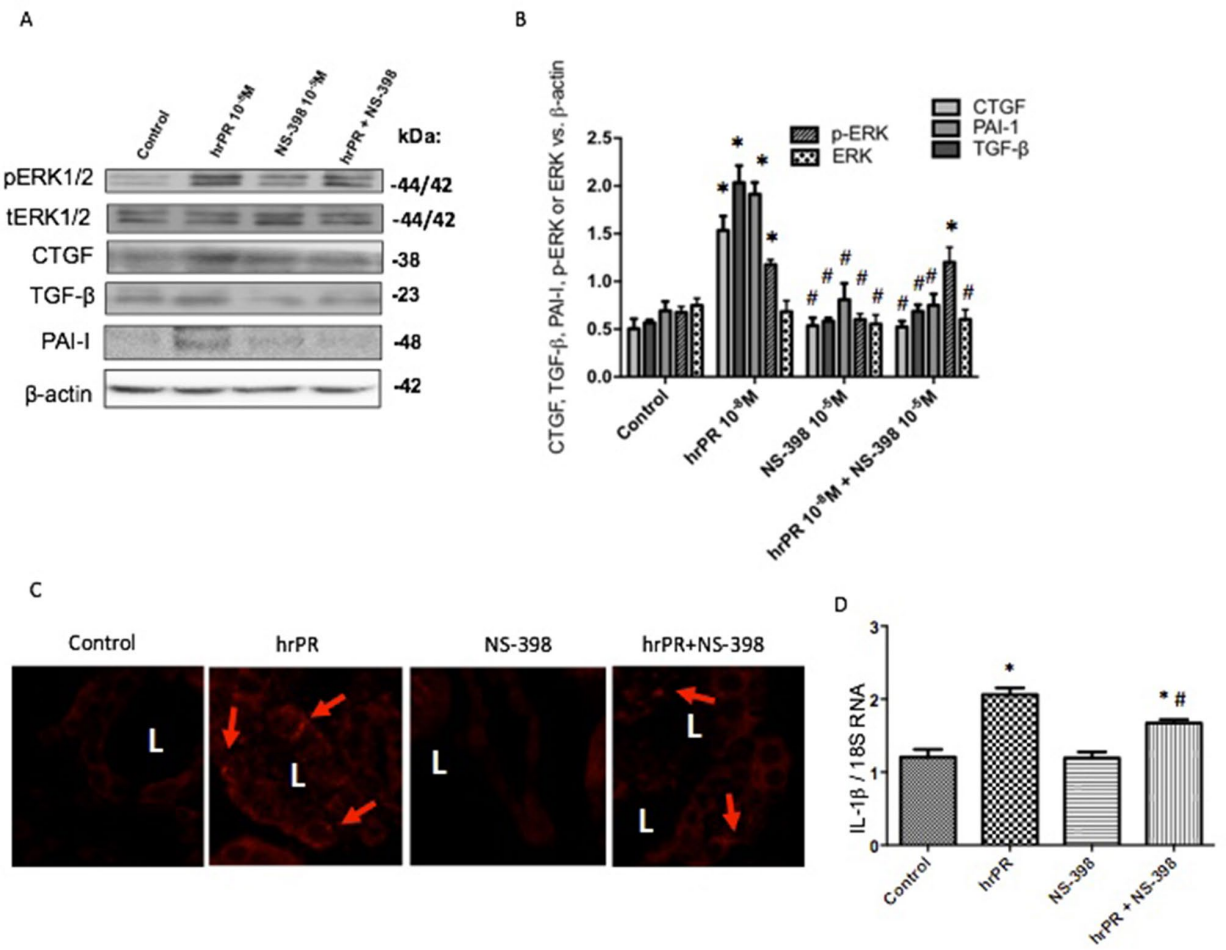
Representative Western blot images **(A)** and quantitation **(B)** of phospho-ERK, total ERK, CTGF, TGF-β, and PAI-I in inner medullary tissues (*n* = 5). *p < 0.05 versus control, #*p* < 0.05 versus hrPR group. **(C)**. Immunofluorescence of alpha smooth muscle actin (α-SMA), a fibrosis-related marker in kidney sections from control mice, saline-infused mice, and mice infused with hrPR. Staining is present in some tubular cells but not in the interstitial cells in renal slides of mice infused with hrPR. A reduced α-SMA staining was evidenced in mice infused with NS-398. **(D)**. IL-1β mRNA relative to 18S mRNA as indicative of inflammatory damage. *p* < 0.05 versus control, ^#^ *p* < 0.05 versus hrPR group.

We tested the effect of chronic infusion of hrPR at a rate of 100 ng/min, *via* an osmotic minipump for 36 h on normal and treated plasma prorenin levels. Using renin enzymatic activity, prorenin was measured in kidney homogenates as the difference between renin activity before and after trypsin activation of prorenin. Estimated plasma prorenin levels were 1,564 ± 109 in controls and 3,514 ± 201 in hrPR-infused groups (*p* < 0.05, *n* = 4). Although PRR has been described at the apical aspect of intercalated CD cells (Gonzalez et al., 2011; Gonzalez et al., 2013), it has also been described at the basolateral membrane (Wang et al., 2016). Physiological parameters such as body weight, kidney weight, urine osmolality, urine flow, Na+ and K+, and estimated GFR are shown in **Table 1**. Prorenin infusion during 36 h slightly reduced Na+ excretion and significantly increased urine osmolality. As shown in **Figure 6**, immunofluorescence studies in kidney slides from control mice showed mostly apical distribution of PRR (in red color and arrows) surrounding the luminal aspect of the CD. Then, we expect that the actions of hrPR might be mediated through blood and filtered hrPR-dependent activation of PRR. **Figure 6E** shows the protocols in mice and the extractions of inner medullary CDs to perform immunoblots and immunofluorescence. Freshly isolated inner medullary CDs (**Figure 6F**) from chronically infused hrPR mice showed augmented fibronectin and collagen I immunofluorescence intensity as compared to controls. NS-398-treated mice did not show changes in immunofluorescence intensity when compared to controls. Mice with COX-2 inhibition treatment and hrPR infusions did not show increase in fibronectin or collagen I staining (**Figures 6G, H** **)**. We next examined the expression profile of phosphorylated and total ERK1/2 and profibrotic genes in renal tissues from mice. As shown in **Figure 7**, the changes in CTGF, PAI-I, and TGF-β expression in inner medullary tissues caused by hrPR infusion were accompanied by increased ERK1/2 phosphorylation (ratio protein/β-actin densitometric values: controls, 0.67 ± 0.01 vs. hrPR, 1.76 ± 0.02, *p* < 0.05). However, despite ERK phosphorylation, the expression of CTGF, PAI-I, and TGF-β was not different from controls in mice infused with hrPR and treated with NS-398. Positive staining for α-SMA was present in some tubular cells but not in interstitial renal cells from mice infused with hrPR. A reduction in the staining was evidenced in mice infused with NS-398 (**Figure 7C**). Finally, we determined levels of IL-1β mRNA relative to 18S mRNA as indicative of inflammatory damage. **Figure 7D** shows a small but significant increase in IL-1β (IL-1β/18S mRNA, hrPR: 2.06 ± 0.09 vs. control: 1.21 ± 0.11, *p* < 0.05) that was partially prevented by COX-2 inhibition (hrPR+NS398: 1.67 ± 0.04 vs. hrPR: 2.06 ± 0.09, *p* < 0.05).

## Discussion

The possible pathological role of PRR has been under intense investigation during the last two decades. Seminal experiments showing that PRR is able to activate prorenin and renin catalytic activity (Nguyen and Burckle, 2004; Nguyen et al., 2004) pointed out its possible role in intratubular and intrarenal Ang I formation with the consequent increase in Ang II levels, contributing to the deleterious effects of Ang II such as vasoconstriction (Nguyen and Contrepas, 2008), antinatriuresis (Nguyen et al., 2002), and profibrotic signals (Clavreul et al., 2011). All these effects are especially relevant in hypertension and kidney disease. The activation of PRR by its agonists is relevant, given the evidence that shows that in diabetes, there are high levels of circulating prorenin (Franken et al., 1992; Chiarelli et al., 2001) and upregulation of the PRR (Huang and Siragy, 2009; Huang and Siragy, 2010). Similarly, it is suggested that high plasma prorenin concentration plays a role in the development of coronary artery disease (Yoshida et al., 2015). Transgenic rats overexpressing PRR show renal tissue damage (Kaneshiro et al., 2006) and elevated blood pressure (Burckle et al., 2006). Additionally, in animal models of diabetes and hypertension, the synthesis and secretion of prorenin and renin are greatly augmented in the principal cells of the CD (Prieto-Carrasquero et al., 2004; Kang et al., 2008; Prieto-Carrasquero et al., 2008), supporting the concept of a local activation of a tubular renin–angiotensin system. Thus, the mechanisms by which PRR may influence local renin–angiotensin system and tissue damage need to be clarified.

**Figure 8:**
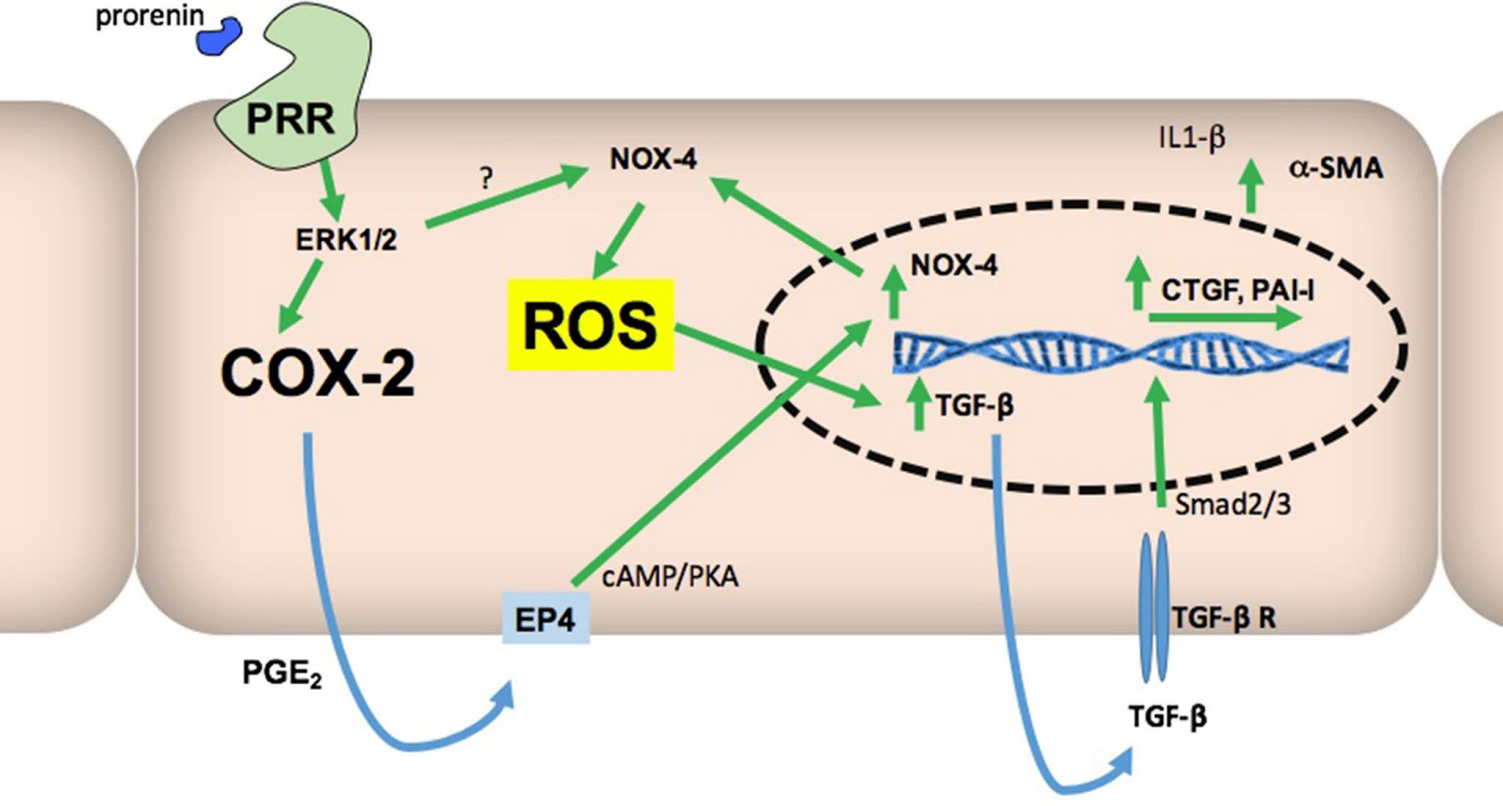
Proposed model to explain how COX-2 might be essential in the stimulatory pathway for ROS production and increased expression of profibrotic genes mediated by PRR activation. Stimulation of PRR by prorenin or renin activates ERK1/2 phosphorylation leading to COX-2 and NOX-4 upregulation. Our data also suggest that upregulation of NOX-4 depends on both the MAPK pathway and activation of EP4 receptor. ROS formation also depends on NOX-4 activity and EP4 signaling, and ROS is responsible for TGF-β upregulation and activation of Smad pathway through TGF-β receptor. This would then lead to CTGF and PAI-I upregulation. Targeting COX-2-mediated prostaglandin E2 (PGE_2_) synthesis may reduce NOX-4 activity and ROS production.

We have previously shown that the activation of PRR increases ROS and profibrotic genes in cultured M-1 CD cell line, which supports the hypothesis that the activation of this receptor may generate renal tissue damage (Gonzalez et al., 2017). However, the exact mechanisms are still unclear. By using PRR’s natural ligand prorenin (recombinant human prorenin) at nanomolar concentrations, we demonstrated that the activation of PRR activates MAPK pathway and upregulates COX-2 and NOX-4. PRR activation also promotes intracellular ROS accumulation and the upregulation of CTGF, TGF-β, and PAI-1. These effects are blunted by pharmacological inhibition of MAPK, NOX-4, and COX-2 enzymatic activities. Importantly, we showed that the antagonism of the E-prostanoid receptor EP4, which is a Gs-coupled receptor (Gs/cAMP/PKA pathway activator), also prevented the upregulation of NOX-4 and profibrotic factors. Interestingly, the phosphorylation of Smad2/3 was prevented by EP4 antagonist, indicating that TGF-β receptor may be not activated due to the impairment of the autocrine actions of TGF-β (**Figure 8**). We also demonstrated that prorenin infusions increase the expression of profibrotic genes and fibronectin and collagen I positive staining in mice isolated CDs. Importantly, the co-treatment with a selective COX-2 inhibitor NS-398 prevented this effect, despite the activation of MAPK pathway (**Figure 7**). This indicates that COX-2 activity, and probably EP4 activation, is necessary for the PRR-dependent upregulation of profibrotic factors and cellular ROS generation.

At micromolar concentrations, PD98059 is a highly selective *in vitro* inhibitor of MEK1 activation and MAPK cascade (Alessi et al., 1995). In our experiment, two different concentrations of the inhibitor were used, based on the IC50 described with respect to the inhibition it generates in ERK 1 (4 μM) and in ERK 2 (50 μM) and in the evidence obtained by different authors previously (Gonzalez et al., 2017). **Figure 2** shows that there is no significant difference between levels of ROS obtained for two concentrations tested; as a result, we continued working with the concentration that was previously established by our group (30 μM). As shown in **Figure 2**, we confirmed our previous studies, which showed that ERK inhibition impairs ROS generation and profibrotic gene expression mediated by PRR stimulation. Next, we proceeded with studying the effects of NOX-4 inhibition in CD cells.

Clavreul et al. demonstrated that in HEK cells that were transfected with a siRNA targeting the PRR, the expression of NOX-4 was prevented as well as the increase in superoxide production, TGF-β, fibronectin, and PAI-1expression (Clavreul et al., 2011). Due to this evidence, we decided to use a pharmacological inhibitor of NOX-4, which is the main isoform with physiological actions in CD cells (Lu et al., 2016). The pharmacological inhibitor GKT 137831 has been described as a dual inhibitor of both NOX-4 and NOX-1 (Green et al., 2012). The effectiveness of GKT 137831 has been demonstrated in cells of the pulmonary vascular wall and cardiac fibroblasts, where it blocks the action of NOX-4 and, thereby, prevents an increase in ROS production (Green et al., 2012). To evaluate its effects on ROS generation in the M-1 CD cell line, we tested three different concentrations, based on concentrations used in the studies of Green et al. (Green et al., 2012). These concentrations were 10, 20, and 30 μM. As seen in **Figure 2**, the addition of this inhibitor prevents ROS induction in the M-1 CD cells at all three concentrations. However, we decided to work with 30 μM, since at this concentration, ROS generation was much closer to the control (**Figure 2**). NOX-4 inhibition was able to prevent the increase in ROS with respect to the basal condition, which indicates in the first instance that the main source of ROS is effectively NOX-4, which is consistent with Clavreul’s evidence (Clavreul et al., 2011). GTK 137831 also prevented the increase in the synthesis of profibrotic proteins **(** **Figure 3** **)**, indicating that the activation of NOX-4 can directly activate the production of profibrotic factors.

Activation of the prostaglandin receptor EP4 increases NOX-4 expression in liver cells. In addition, overexpression of COX-2 leads to higher NOX-4 levels and ROS content, while inhibition of the enzyme leads to decreased NOX-4 levels and ROS content (Sancho et al., 2011). In turn, ROS are involved in TGF-β and Smad signaling (Lafon et al., 1996; Chiu et al., 2001), which induces fibrotic factors CTGF and PAI-I (Clarkson et al., 1999; Kilari et al., 2018). The ERK pathway can enhance Smad activity. On the other hand, ERK inhibition reduces TGF-β1-stimulated Smad phosphorylation as well as collagen production and promoter activities, suggesting that ERK activity is necessary for an optimal response to TGF-β1 (Hayashida et al., 2003). Although we observed a reduction in ROS production and profibrotic factors in hrPR-treated cells, the suppression was not complete. This incomplete suppression indicates that the activity of endogenous NOX-4 may be induced by MAPK pathway independent of COX-2-mediated PGE_2_ and EP4 activation. Furthermore, it is possible that low levels of ROS might function in various pathophysiological processes, contributing to the activation of transcription factors leading to induction of profibrotic factors as well (Sedeek et al., 2013a; Sedeek et al., 2013b). We have reported that antioxidants prevented the increase in profibrotic factors, indicating an evident role of ROS as a signaling agent in a pathophysiological process (Gonzalez et al., 2017).

Our data were corroborated *in vivo* using chronic infusions of human recombinant prorenin during 36 h. We observed an increased expression of profibrotic genes in isolated CDs from treated mice through Western blot analysis of pERK and total ERK, CTGF, TGF-β, and PAI-I and by immunofluorescence analysis of fibronectin and collagen I expression (**Figures 6** and **7**) We also observed α-SMA staining in mice with hrPR infusions, which was less evident in mice treated with COX-2 inhibitor. It is possible that recombinant prorenin infusion increased profibrotic protein expression through direct interactions with apical PRR by filtration from plasma and having access to the distal tubular lumen or by having access to basolateral PRR through the blood.

Despite the evidence of the role of NOX-4 and ERK pathway in renal fibrosis, little is known about their interactions with COX-2. PRR and COX-2 are co-located in CD cells (Gonzalez et al., 2013), which suggests that they could be functionally related. Kaneshiro et al. demonstrated in 2006 that the overexpression of human PRR in rats resulted in an over-regulation of COX-2 in renal cortex, which contributed to the generation of tubular damage, due to the inflammation mediated by prostaglandins (Kaneshiro et al., 2006). In addition to this evidence, it is known that the activation of MAPK activates COX-2, which consequently leads to production of PGE_2_ in the CD. Furthermore, we have recently published evidence of the participation of PGE_2_, synthesized by COX-2, in the regulation of prorenin. Prorenin causes further increases in COX-2 expression, generating transient COX-2-prorenin positive feedback (Salinas-Parra et al., 2017). As mentioned before, the relationship between COX-2 and NOX-4 observed in hepatocytes (Sancho et al., 2011) suggests that a similar system regulated by positive feedback may be present in CD cells (**Figure 8**).

In summary, our results indicate that the induction of ROS, TGF-β, and profibrotic factors CTGF and PAI-I occurs through PRR-dependent activation of MAPK and NOX-4. Additionally, it depends on intact COX-2 activity that leads to PGE_2_-dependent activation of EP4 receptor and TGF-β receptor-dependent Smad pathway activation. Site-specific pharmacological inhibition of COX-2 in the CD may help to prevent tubular damage during states of activation of intratubular renin–angiotensin system, such as hypertension and diabetes.

## Ethics Statement

The Institutional Animal Care and Use Committees of the Pontificia Universidad Catolica de Valparaiso approved all animal protocols.

## Author Contributions

AG, CR-M, MK, and QMN performed the experiments, analyzed the data and provided the final version of the manuscript.

## Funding

This study was funded by DI Grant Nº 0.39.307/2018 Pontificia Universidad Católica de Valparaíso, Chile and Fondo Nacional de Desarrollo Científico y Tecnológico (FONDECYT) 1191006.

## Conflict of Interest Statement

The authors declare that the research was conducted in the absence of any commercial or financial relationships that could be construed as a potential conflict of interest.

The handling editor declared a shared affiliation, though no other collaboration, with the authors CR-M and AG, at the time of review.
